# Intrahepatic Cholangiocarcinoma Cells Promote Epithelial-mesenchymal Transition of Hepatocellular Carcinoma Cells by Secreting LAMC2

**DOI:** 10.7150/jca.55627

**Published:** 2021-04-19

**Authors:** Wenda Cen, Jiandong Li, Chenhao Tong, Weiguang Zhang, Yunfeng Zhao, Baochun Lu, Jianhua Yu

**Affiliations:** 1 Shaoxing University School of Medicine, Shaoxing, China.; 2 Department of Hepatobiliary Surgery, The First Affiliated Hospital of Shaoxing University, Shaoxing, China.; 3 Department of Hepatobiliary Surgery, Shaoxing People's Hospital (Shaoxing Hospital, Zhejiang University School of Medicine), Shaoxing, China.; 4 Department of Molecular Medicine and Clinical Laboratory, Shaoxing Second Hospital, Shaoxing, China.; 5 Department of Pharmacology, Toxicology & Neurosciences, LSU Health Sciences Center, Shreveport, LA, USA.

**Keywords:** Hepatocellular carcinoma, Intrahepatic Cholangiocarcinoma, Combined hepatocellular cholangiocarcinoma, LAMC2, EMT.

## Abstract

Hepatocellular carcinoma and intrahepatic cholangiocarcinoma cells are common primary hepatic tumor cells in the liver. Combined hepatocellular cholangiocarcinoma (CHC) contains both hepatocellular carcinoma cells and intrahepatic cholangiocarcinoma cells in one tumor lesion and these tumors show poor prognosis. Here we examined the potential interaction between hepatocellular carcinoma cells and intrahepatic cholangiocarcinoma cells using cell culture studies. The results showed that culture supernatant from intrahepatic cholangiocarcinoma cells induced endothelial-mesenchymal transition and facilitated the migration and invasion of hepatocellular carcinoma cells, although it did not accelerate the proliferation of hepatocellular carcinoma cells. Furthermore, culture supernatant from intrahepatic cholangiocarcinoma cells increased the chemoresistance of hepatocellular carcinoma cells. Laminin subunit gamma 2 (LAMC2) was detected in the culture supernatant of intrahepatic cholangiocarcinoma cells but not in that of hepatocellular carcinoma cells. Using established LAMC2 knockout intrahepatic cholangiocarcinoma cells, our results demonstrated that intrahepatic cholangiocarcinoma cells promoted the epithelial-mesenchymal transition of hepatocellular carcinoma cells through secreting LAMC2. Our results have revealed a novel mechanism of interaction between intrahepatic cholangiocarcinoma cells and hepatocellular carcinoma cells, which may provide new insight into developing effective treatments for CHC.

## Introduction

Hepatocellular carcinoma (HCC) is the most common type of primary liver cancer, accounting for approximately 80% of all cases, and intrahepatic cholangiocarcinoma (ICC) is the second most common liver cancer, accounting for about 15% of cases 1. HCC is comprised of hepatocellular carcinoma cells (HCC cells) and ICC is comprised of intrahepatic cholangiocarcinoma cells (ICC cells). Besides, there is a form of primary liver carcinoma contains a mixture of HCC cells and ICC cells, namely combined hepatocellular cholangiocarcinoma (CHC), with an incidence varying from 1.0% to 14.3% of primary liver cancer in different studies 1-5. Although the number of CHC cases is relatively small, it is difficult to diagnose before surgery and has a dismal prognosis 1, 6. The data from a long-term prognosis study of liver cancer indicates that CHC has a significantly worse prognosis than HCC and ICC even after curative resection 1. Given the special histological characteristics of CHC, we speculated that the unique cellular component of CHC could contribute to its dismal prognosis.

Epithelial-mesenchymal transition (EMT), a critical process in which cancer cells gain enhanced invasion, migration, and metastasis abilities, has been associated with poor prognosis 7. Cells undergoing EMT show loose or no interactions among cells, lost the expression of certain adhesion molecules such as E-cadherin, and acquire the expression of mesenchymal-associated markers such as vimentin and snail 7, 8. It has been shown that EMT is an important contributing factor to the development and progression of liver cancer 9, 10.

Laminins, a large family of extracellular matrix proteins, are comprised of three different chains (α, β and γ). Laminin-332 is a well-studied member of laminins and has been reported as an important regulator in the process of oncogenesis 11, 12.* LAMC2* encodes the γ2 chain of laminin-332 and is an important EMT-associated gene in various cancers including bladder cancer, colorectal cancer, lung cancer, and cholangiocarcinoma 13-16. Early studies have reported that LAMC2 exists in the tumor microenvironments and influences the invasion and migration of cancer cells 17, 18.The tumor microenvironment plays a critical role in influencing the biological behavior of cancer cells. We speculate that there might be interactions between the HCC and ICC components in CHC, which contribute to the poor prognosis of CHC. For this purpose, we incubated HCC cells with culture supernatant from ICC cells, and examined possible mechanisms of action.

## Material and Methods

### Reagents

Cisplatin and doxorubicin were purchased from Cayman Chemical (Ann Arbor, MI, USA). Antibodies against β-actin (sc‑47778), LAMC2 (sc‑28330), E-cadherin (sc‑71009), Vimentin (sc‑80975) and Snail (sc‑271977) were purchased from Santa Cruz Biotechnology, Inc. (Dallas, TX, USA).

### Cell culture

The HepG2 and SNU-449 cell lines were obtained from the American Type Culture Collection (Manassas, VA, USA). The HCCC-9810 cell line and PLC/PRF/5 cell line were obtained from the Chinese Academy of Sciences Shanghai Branch Cell Bank (Shanghai, China). The HuCCT1 cell line was purchased from the RIKEN BioResource Center (Ibaraki, Japan). HepG2, SNU-449 and PLC/PRF/5 cell lines are hepatocellular carcinoma cells, whereas HCCC-9810 and HuCCT1 cell lines are intrahepatic cholangiocarcinoma cells. HepG2, SNU-449, PLC/PRF/5, HCCC-9810 and HuCCT1 cells were cultured in RPMI 1640 medium (Gibco, Thermo Fisher Scientific, Inc.) supplemented with 10% fetal bovine serum (FBS, Gibco, Thermo Fisher Scientific, Inc.), 100 IU/ml penicillin and 100 µg/ml streptomycin. After informed consent was obtained from patients and the tissue acquisition protocol was approved by the Ethics Committee of Shaoxing People's Hospital, liver explant tissue from a female patient with hepatic hemangioma without other hepatic diseases was obtained. Human cholangiocytes were isolated from normal liver tissue samples by magnetic bead-purification (Miltenyi Biotec, Inc.), and were cultured with DMEM/F12 medium (Gibco, Thermo Fisher Scientific, Inc.) as previously described 19.

### Preparation of cell culture supernatants

Cells were plated in a 100-mm dish at 40%-50% confluence, and the culture medium was replaced with fresh RPMI 1640 medium after cells attached to the plate. Twenty-four hours later, culture supernatant was collected and filtrated through a 0.22 µm filter (Millipore, Merck KGaA, MA, USA). The filtrated culture supernatants from HepG2, SNU-449, PLC/PRF/5, HCCC-9810, HuCCT1 cells, and cholangiocytes were used for subsequent experiments.

### Colony formation assay

HepG2, PLC/PRF/5 and SNU-449 cells were seeded in a 12-well plate at 100 cells per well. After cells being attached, the culture medium was replaced with filtrated culture supernatants from either the same cell line (e.g. HepG2 clones were cultured with culture supernatants from HepG2 cells), cholangiocarcinoma cell lines, or cholangiocytes.

The culture supernatants were replaced every 3 days. After 14 days, cells were fixed in 4% paraformaldehyde for 15 min. After washing, cells were stained with 0.005% crystal violet solution for 1 h. The plates were aspirated, washed, and allowed to air dry. The morphology of cell colonies was observed using light or fluorescence microscopy.

### Western blot analysis

Protein samples (20 µg) of whole-cell lysate were separated on a 10% SDS-PAGE gel and transferred onto a polyvinylidene fluoride (PVDF) membrane. The membrane was blocked and incubated with a primary antibody, followed by incubation with a horseradish peroxidase-conjugated secondary antibody (Jackson ImmunoResearch Inc., West Grove, PA, USA). Immunoreactive bands were visualized using a chemiluminescence solution (Genesee Scientific, El Cajon, CA, USA). Experiments were repeated three times. β-actin was used as the loading control for cell lysis. The protein bands were quantified using the ImageJ software.

For analysis of culture supernatants, the culture medium was centrifuged and filtrated with a 0.22 µm filter to remove cells. Supernatant samples (30 µl) were combined with 4 x loading buffer (10 µl), denatured, separated by SDS-PAGE, and analyzed as described above. Ponceau S staining of the PVDF membrane was used as the loading control for electrophoresis of the culture medium.

### Wound healing assays

HepG2 and SNU-449 cells (2×10^4^) were seeded in 96-well plates (Essen BioScience, Ann Arbor, MI, USA) and cultured overnight. Scratches were introduced by a Woundmaker (Essen BioScience). Wells were washed twice with serum-free medium, and different culture supernatants were added. After 48 h, images were obtained and analyzed. Six replicate wells from each cell treatment were analyzed.

### Transwell invasion assays

After culture overnight in serum-free medium, cells (1×10^4^) in 0.1 ml serum-free culture supernatant were placed into the upper chamber of a transwell insert (Corning, NY, USA) coated with Matrigel (BD Biosciences, San Jose, CA, USA). 0.5 ml culture supernatant with 10% FBS was added to the lower chamber. Twenty-four hours later, cells that migrated to the other side of the insert were fixed with 4% paraformaldehyde for 15 min following by crystal violet staining. These experiments were repeated three times.

### Cell proliferation assays

Equal numbers of cells were seeded in 96-well plates and incubated with different culture supernatants. Cell growth was determined using the 3-[4,5-dimethylthiazol-2-yl]-2, 5-diphenyltetrazolium bromide assay (MTT) assay (Sigma, St. Louis, MO, USA). One-tenth volume of MTT diluted in serum-free medium was added to each well at different time points, and the plates were incubated at 37˚C for 4 h. Formazan crystals were dissolved in DMSO, and the absorbance at 595 nm was measured using a spectrometer (Bio-Rad, Philadelphia, PA, USA). Six replicate wells from each cell type were analyzed.

### EdU proliferation assays

HepG2 cells (2×10^3^) were seeded into 6-well plates and allowed to attach overnight. The culture medium was replaced with different culture supernatants containing 10 µM EdU (Beyotime, Nanjing, China) for 2 h. Cells were washed with PBS and fixed with cold 4% paraformaldehyde. After treating with 0.3% TritonX-100, washing with PBS and blocking, cells were incubated with Azide 555 and Hoechst 33342 (Beyotime). Fluorescence was immediately detected using a Nikon microscope (Tokyo, Japan). Experiments were repeated three times.

### Drug sensitivity assays

Cisplatin and doxorubicin were used to evaluate the effect of different culture supernatants on the chemotherapy sensitivity of the cells. Cells (4×10^3^) were seeded into 96-well plates and allowed to attach overnight. Cells were then treated with the different culture supernatants containing various concentrations of cisplatin and doxorubicin. Twenty-four hours later, viable cells were determined using the MTT assay as described above.

### Cell apoptosis analysis

HepG2 cells were treated with different culture supernatants for 24 h. Cisplatin was added to culture supernatants at a final concentration of 1 µg/ml. Four hours later, cells were collected and stained with Annexin V-FITC and propidium iodide (PI) (Becton-Dickinson, Franklin, NJ, USA) following the manufacturer's instructions. Apoptotic cells were quantified using a flow cytometer (Beckman Coulter, Fullerton, CA, USA). Experiments were repeated three times.

### Caspase 3 activity detection

Caspase 3 activity in HepG2 cells was detected using the Caspase 3 Activity Assay Kit (Beyotime) following the manufacturer's protocol. The absorbance was measured at 405 nm, and the relative activity of Caspase 3 was calculated.

### Establishment of LAMC2 stable knockout (KO) clones

HuCCT1 cells were transfected with the Laminin γ-2 CRISPR/Cas9 KO Plasmid (Santa Cruz Biotechnology), which consists of a pool of three plasmids, encoding the Cas9 nuclease and a target-specific 20 nt guide RNA. At 72 h post-transfection, cells were sorted using flow cytometry by expressing green fluorescence (GFP). The selected clones were amplified and Western blot analysis was performed to select LAMC2 KO clones.

### Clinical tissue samples

Tissue samples were obtained from patients with liver tumors who had a definite pathological diagnosis after surgery at the Shaoxing People's Hospital from January 2013 to February 2018. Informed consent was obtained from patients and the tissue acquisition protocol was approved by the Ethics Committee of Shaoxing People's Hospital. Definite HCC components and ICC components were simultaneously found in the CHC samples by pathological examination. There was no significant difference between the general characteristics of HCC patients and those of CHC patients, including gender and age. Fresh tissues were frozen in liquid nitrogen and used for protein extraction.

### Statistical analysis

Data were presented as means ± SD. Statistical significance between the two groups was determined using the Student's t-test. One-way ANOVA followed by the Tukey-Kramer adjustment was used to examine differences among multiple groups. p < 0.05 was considered to be significant. All statistical analyses were conducted using SPSS 11.0.

## Results

### Culture supernatant from intrahepatic cholangiocarcinoma cells induces EMT in hepatocellular carcinoma cells

**Figure 1:**
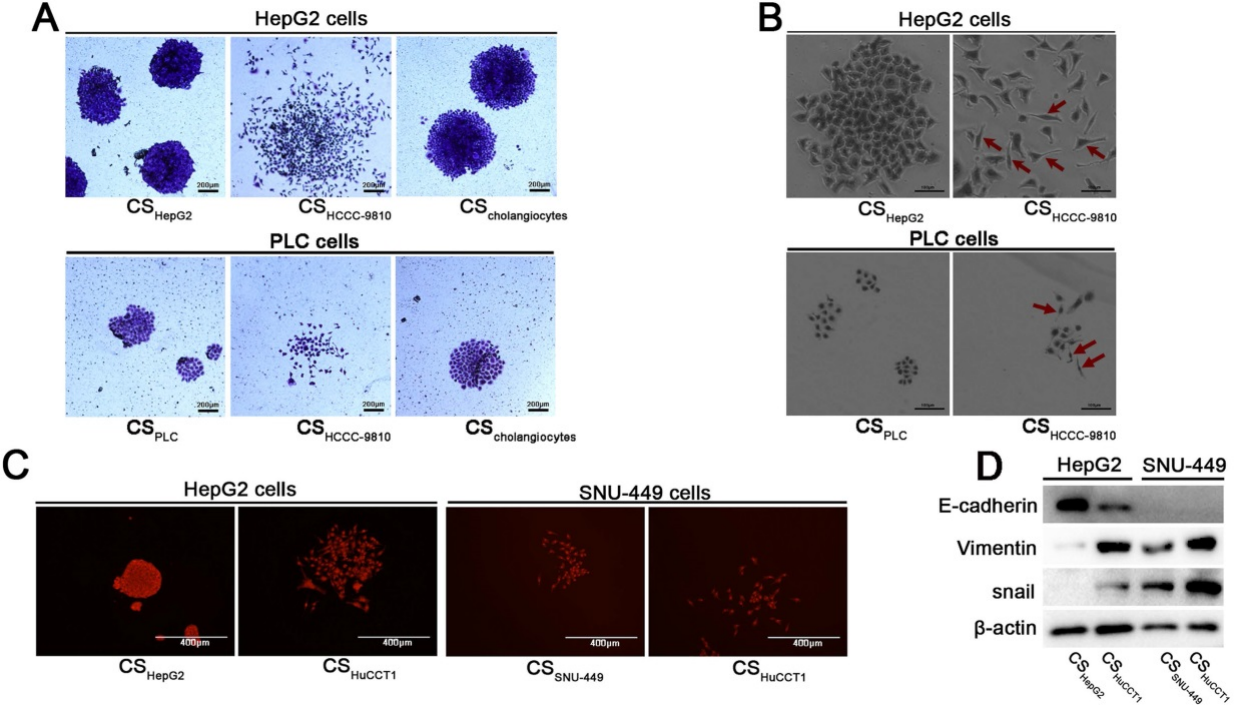
** Culture supernatant from ICC cells induces EMT in HCC cells. (A)** Colony formation assays of HepG2 and PLC/PRF/5 cells incubated with different culture supernatants. Cells were stained with crystal violet and observed by light microscopy (scale bars: 200μm). **(B)** The morphology of HepG2 cells incubated with different culture supernatants (scale bars: 100μm). Red arrow: HepG2 cells with a fusiform shape. **(C)** Colony formation assays of HepG2 and SNU-449 cells incubated with culture supernatants from HuCCT1 or HCC cells. These cells were stained with crystal violet and observed using fluorescence microscopy (scale bars: 400μm). **(D)** Detection of the expression levels of E-cadherin, Vimentin, and Snail in HCC cells incubated with different culture supernatants. CS: culture supernatants; PLC cells: PLC/PRF/5 cells.

To examine whether ICC cells affect the behavior of HCC cells, we performed clonal formation assays using HepG2 HCC cells incubated with the culture supernatant of HCCC-9810 ICC cells. Intriguingly, the HepG2 cells grown in HCCC-9810 culture supernatant showed expansive and discrete morphology (Fig. 1A and 1B). These clones were distinctly different from the HepG2 clones cultured in the supernatant of HepG2 cells: they did not form solid (island-like) colonies (Fig. 1A) and the single-cell had a fusiform appearance, which was different from the polygon shape of HepG2 cells (Fig. 1B). Similar cellular morphology was also observed in PLC/PRF/5 HCC cells after treated with the culture supernatant of HCCC-9810 cells (Fig. 1A and 1B).

To confirm these observations were not unique to HCC cells in HCCC-9810 ICC cell culture supernatant, similar studies were performed using other ICC cells and HCC cells. When HepG2 cells were treated with the culture supernatant of HuCCT1 cells, similar cell morphology was observed (Fig. 1C). SNU-449 cells, an invasive HCC cell line, tend to form loose clones compared to HepG2 cells. Similarly, SNU-449 cells grew more sparsely when cultured in the supernatant of HuCCT1 cells (Fig. 1C).

The lack of clear apical and lateral membranes and loose or no interactions among cells are the morphological characteristics of EMT 8. Based on the observed morphological changes described above, the molecular markers for EMT were examined. The epithelial marker E-cadherin was found to be downregulated in HepG2 cells incubated with culture supernatant from ICC cells compared with that incubated with culture supernatant from HCC cells (Fig. 1D). Conversely, mesenchymal-associated proteins Vimentin and Snail were upregulated in HepG2 and SNU-449 cells treated with culture supernatants from ICC cells (Fig. 1D). These results indicate that ICC cells create a microenvironment that induces EMT of HCC cells.

### Culture supernatant of intrahepatic cholangiocarcinoma cells promotes the migration and invasion of hepatocellular carcinoma cells

**Figure 2:**
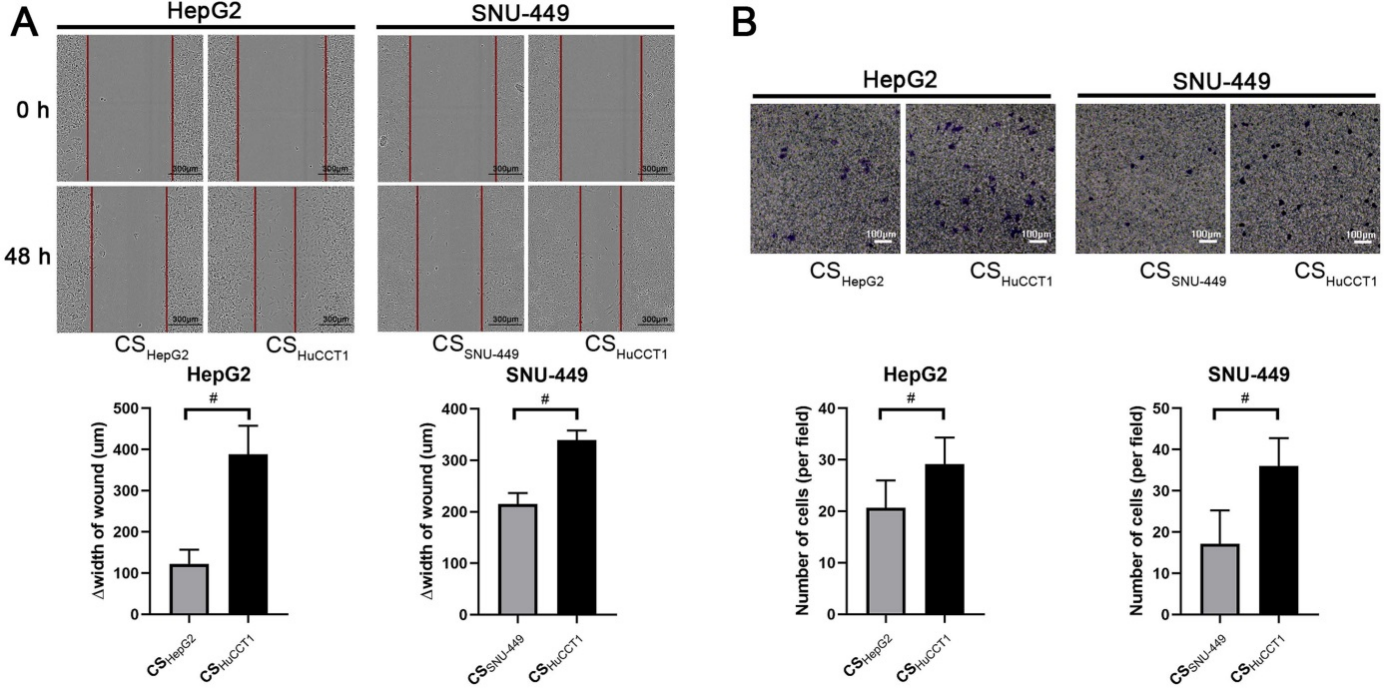
** Culture supernatant from ICC cells promotes the migration and invasion of HCC cells. (A)** Wound healing (scale bars: 300μm) and **(B)** Transwell invasion assays (scale bars: 100μm) of HCC cells incubated with different culture supernatants. △width of wound=width of wound at the start (0 h) - width of wound at the end (48 h). CS: culture supernatants. #*P* < 0.05, significant differences between the two groups.

The key biological significance of EMT for cancer cells is acquiring migration ability and facilitating cancer cell invasion. We next evaluated migration and invasion of HCC cells with or without adding culture supernatant of ICC cells. In wound healing assays, the culture supernatant of HuCCT1 cells induced rapid wound healing in HCC cells compared with the culture supernatant of HCC cells (HepG2 and SNU-449) (Fig. 2A). Furthermore, cells with a fusiform appearance showed increased mobility in the wound area (Fig. 2A). The results from the transwell assay also showed that culture supernatant from HuCCT1 cells enhanced the invasion ability of HepG2 and SNU-449 cells (Fig. 2B).

### Culture supernatant of intrahepatic cholangiocarcinoma cells does not accelerate the proliferation of hepatocellular carcinoma cells

**Figure 3:**
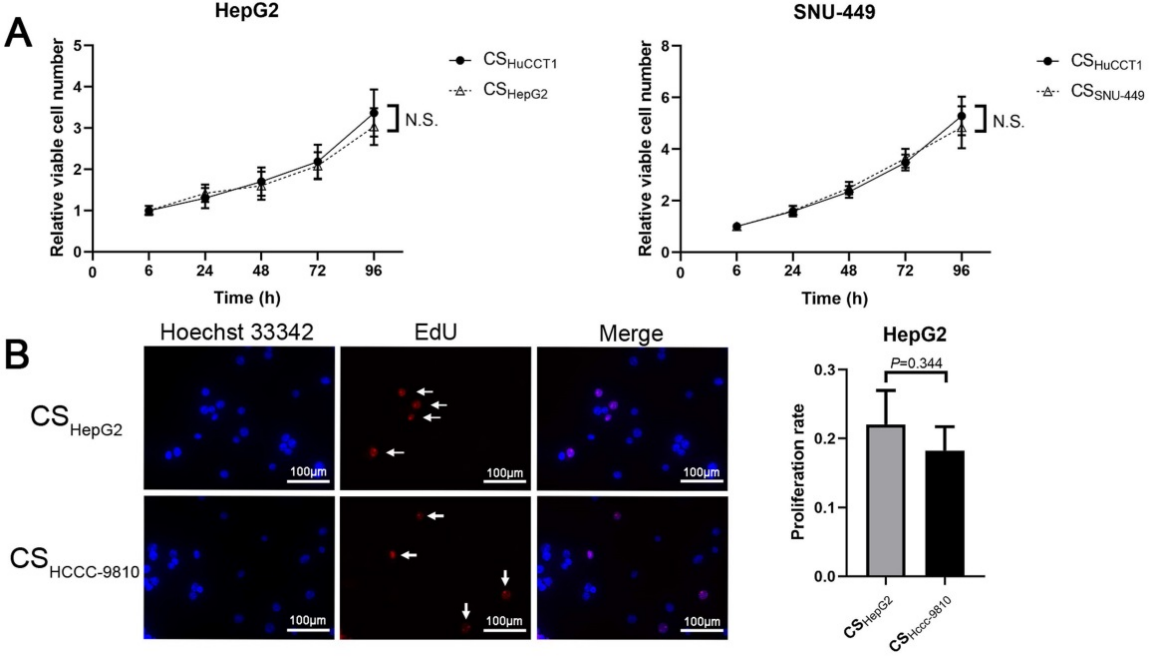
** Culture supernatant from ICC cells does not accelerate the proliferation of HCC cells. (A)** Cell proliferation and **(B)** EdU assays of HCC cells incubated with different culture supernatants (scale bars: 100μm). CS: culture supernatants. N.S. = non-significant. White arrow: EdU-positive cells.

We next studied whether culture supernatant from ICC cells would accelerate cell proliferation of HCC cells. The results showed no significant difference between the growth rates of HCC cells in culture supernatant from HuCCT1 cells and in that from HCC cells (Fig. 3A). Results from EdU-labeling assays also showed that the culture supernatant from HCCC-9810 cells did not accelerate the proliferation of HepG2 cells (Fig. 3B).

### Culture supernatant of intrahepatic cholangiocarcinoma cells enhances the chemoresistance of hepatocellular carcinoma cells

**Figure 4:**
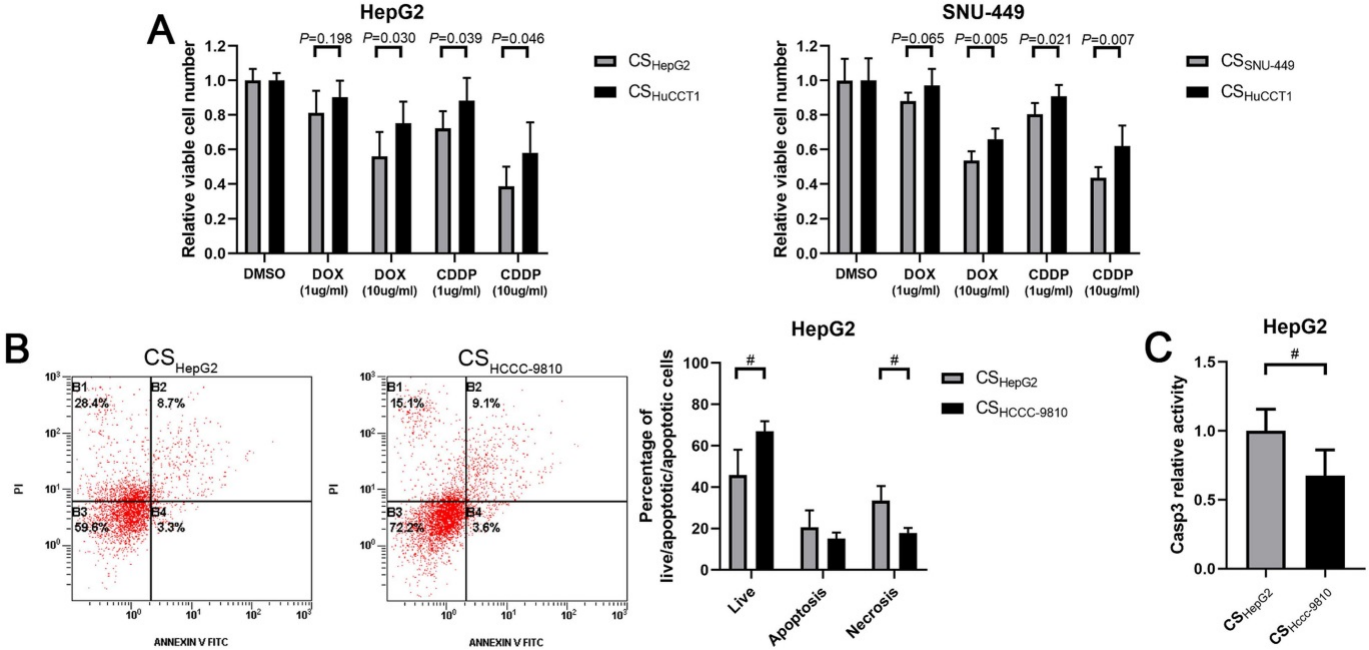
** Culture supernatant from ICC cells enhances the chemoresistance of HCC cells. (A)** Comparison of chemosensitivity of HCC cells incubated with different culture supernatants. **(B)** Detection of apoptosis using flow cytometry in HepG2 cells incubated with different culture supernatants and cisplatin. **(C)** Detection of caspase 3 activity in HepG2 cells incubated with different culture supernatants and cisplatin. CS: culture supernatants. #*P* < 0.05, significant differences between the two groups. CDDP: cisplatin. DOX: doxorubicin.

Chemoresistance is a critical obstacle to the successful treatment of cancers. We next studied the response of HCC cells cultured in ICC cell culture supernatant to chemo drugs. Results from drug sensitivity assays showed that HepG2 cells incubated with culture supernatant from HuCCT1 cells became more resistant to cisplatin and doxorubicin treatments compared to the cells incubated with culture supernatant from HepG2 cells (Fig. 4A). A similar result was also observed in SNU-449 cells treated with culture supernatant from HuCCT1 cells (Fig. 4A).

Apoptotic cell death was detected in these HCC cells. Results from the Annexin V staining assay showed that less cisplatin-induced cell death in HepG2 cells incubated with culture supernatant from HCCC-9810 cells, compared to the cells incubated with culture supernatant from HepG2 cells (Fig. 4B).

Similarly, the activity of Caspase 3 induced by cisplatin treatment was significantly lower in HepG2 cells incubated with culture supernatant from ICC cells compared to the cells incubated with culture supernatant from HCC cells (Fig. 4C).

### Intrahepatic cholangiocarcinoma cells promote EMT of hepatocellular carcinoma cells through secreting LAMC2

**Figure 5:**
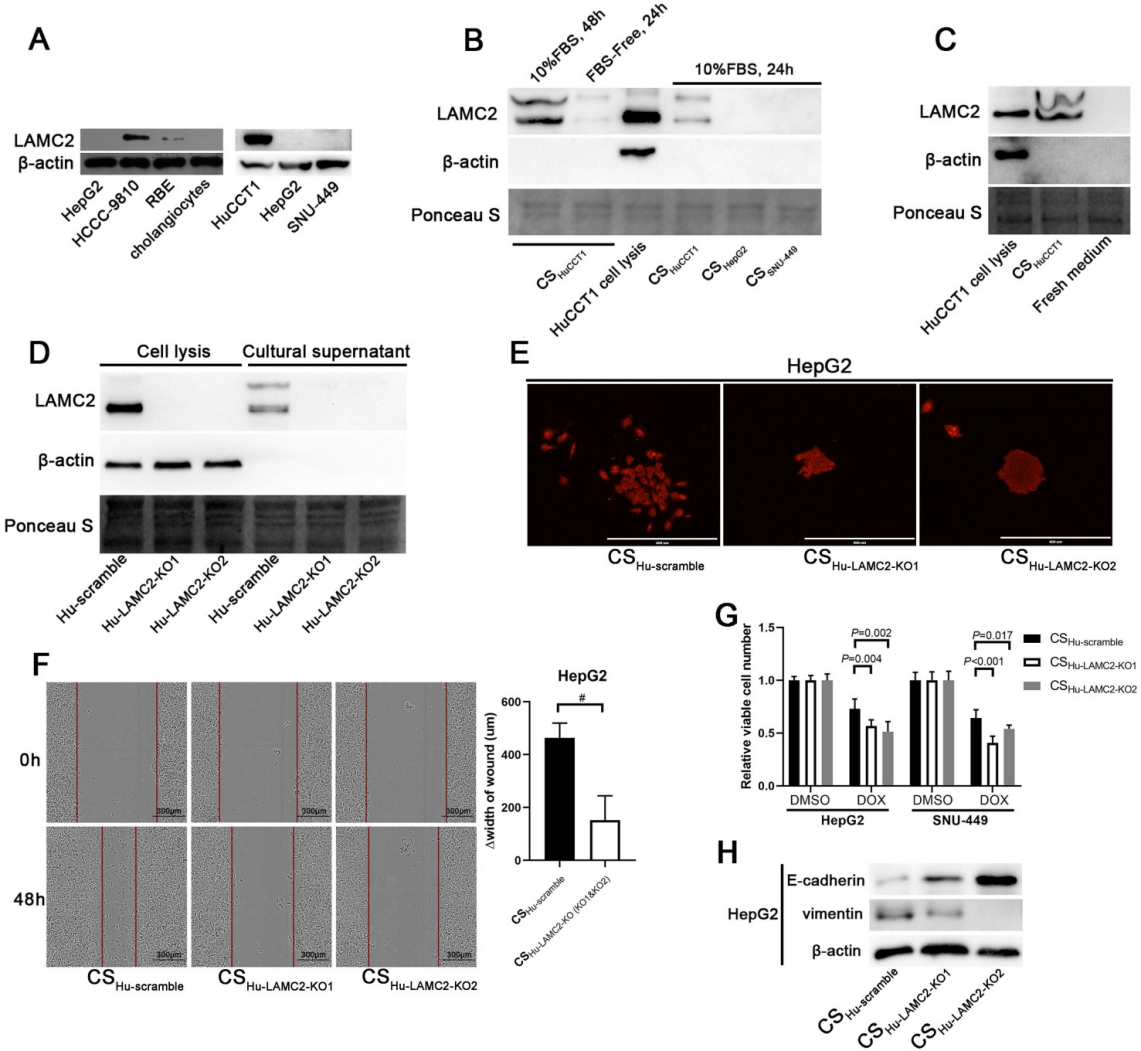
** ICC cells induce EMT of HCC cells through secreting LAMC2. (A)** Detection of LAMC2 in different ICC and HCC cells. **(B)** Detection of LAMC2 in different culture supernatants. The samples were loaded in the same volume (30 µl) and ponceau S was used as the loading control. The cell lysate of HuCCT1 was used as a positive control. β-actin was used to detect cell lysis. Detection of LAMC2 in the fresh medium **(C)** and LAMC2 knockout HuCCT1 cells **(D)**.** (E)** Colony formation assays of HepG2 cells incubated with culture supernatants from LAMC2 knockout HuCCT1 cells (scale bars: 400μm). **(F)** Wound healing assays (scale bars: 300μm) of HepG2 cells incubated with culture supernatants from LAMC2 knockout HuCCT1 cells. #*P* < 0.05, compared to culture supernatants from scramble clone. **(G)** Chemosensitivity of HCC cells incubated with culture supernatants from LAMC2 knockout HuCCT1 cells. **(H)** Detection of the expression levels of E-cadherin and Vimentin in HCC cells incubated with culture supernatants from LAMC2 knockout HuCCT1 cells. CS: culture supernatants.

The tumor microenvironment plays a critical role in influencing the biological behavior of cancer cells, including EMT. A previous study reported that HCC cells showed a similar morphological change when treated with laminin-332 18. Given that *LAMC2* encodes the most important chain of laminin-332 and it has been identified as a poor prognostic marker in some cancers 20, 21, the protein expression levels of LAMC2 in different ICC and HCC cells were evaluated using Western blot analysis. As shown in Fig. 5a, LAMC2 was barely detected in HCC cell lines including HepG2 and SNU-449, but it was highly expressed in ICC HCCC-9810 and HuCCT1 cells (Fig. 5A).

To determine whether LAMC2 is secreted into the medium, we performed Western blot analysis using culture supernatants. LAMC2 was detected in the culture supernatant of HuCCT1 cells, while undetectable in the samples of HCC cell lines (Fig. 5B). As a control, a fresh culture medium with 10% FBS was also analyzed and LAMC2 was undetectable in these samples (Fig. 5C). These results indicate that LAMC2 is secreted by ICC cells into the culture supernatant.

To evaluate whether ICC cells promote EMT of ICC cells depending on LAMC2, LAMC2 KO HuCCT1 cells were established and LAMC2 was undetectable in culture supernatant from these KO cells (Fig. 5D). Culture supernatant from LAMC2 KO HuCCT1 cells was then used to treat HCC cells. Results from the colony formation assay showed solid colonies in HepG2 cells incubated with culture supernatant from LAMC2 KO HuCCT1 cells, different from cells incubated with culture supernatant from scramble control cells (Fig. 5E). Moreover, results from the wound healing assay in HepG2 cells also showed slowing migration when cells were incubated with culture supernatant from LAMC2 KO HuCCT1 cells (Fig. 5F). Results from drug sensitivity assays showed that HepG2 and SNU-449 cells incubated with culture supernatant from scramble HuCCT1 cells were more resistant to doxorubicin treatment compared to the cells incubated with that from LAMC2 KO HuCCT1 cells (Fig. 5G). We further observed that higher E-cadherin and lower Vimentin expression in these KO samples (Fig. 5H). These results indicate that LAMC2 mediates at least partially, the activity of the microenvironment of ICC cells in promoting EMT of HCC cells.

### High expressed LAMC2 is associated with mesenchymal phenotype in CHC tissues

**Figure 6:**
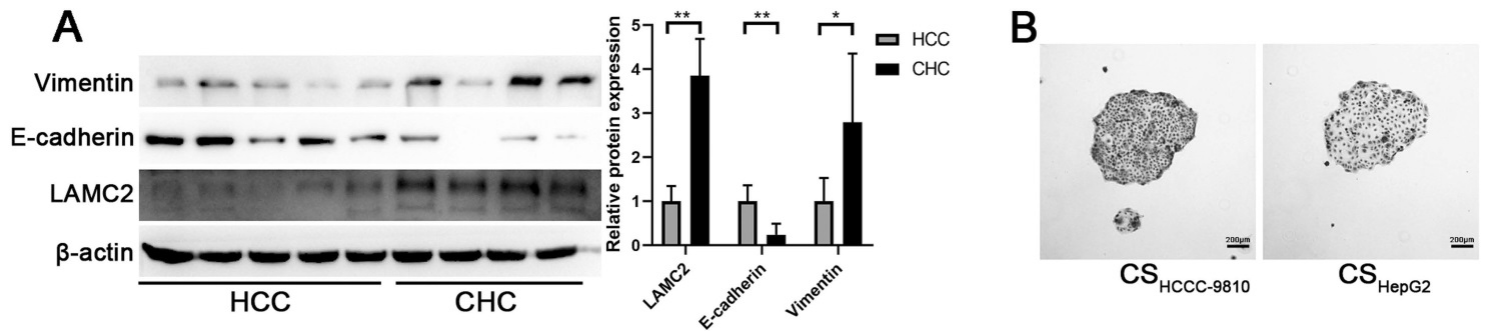
** (A)** Detection of the expression levels of LAMC2, E-cadherin, and Vimentin in human HCC and CHC tissue samples. #*P* < 0.05; ##*P* < 0.01, significant differences between the two groups. **(B)** Colony formation assays of HCCC-9810 cells incubated with different culture supernatants (scale bars: 200μm).

The expression levels of LAMC2 between CHC and HCC tissue samples were compared. Results from Western blot analysis showed that LAMC2 protein levels were significantly higher in CHC samples than those in HCC samples (Fig. 6A). Higher Vimentin and lower E-cadherin expression levels were also found in CHC samples compared to HCC samples. These results demonstrate that the levels of LAMC2 expression are positively associated with the mesenchymal phenotype in clinical CHC samples.

## Discussion

CHC is defined as mixed hepatocellular carcinoma-cholangiocarcinoma, hybrid hepatocellular carcinoma-cholangiocarcinoma, or combined liver and bile duct carcinoma 4, 22. Although it has been speculated that CHC may arise from hepatic progenitor cells, there is no solid evidence demonstrating the common origin of different components of CHC 23, 24. CHC contains components of both HCC and ICC, therefore, CHC shows characteristics similar to HCC, such as viral hepatitis B infection and elevated alpha fetal protein (AFP), as well as those similar to ICC, such as elevated carbohydrate antigen 19-9 and lymphatic metastasis 25. Notably, although CHC is a rare type of tumor, it has a worse prognosis and more aggressive behavior compared to HCC and ICC 1, 4, 26, 27. Given the cellular components of CHC, we speculated that there might be interactions between the HCC and ICC components, which contribute to the poor prognosis of CHC. To study this possibility, we used culture supernatant from ICC cells to treat HCC cells, and that from HCC cells to treat ICC cells. As shown in the results (Fig. 1), morphological features of EMT are observed in HCC cells incubated with culture supernatant from ICC cells. No remarkable morphological change is observed in ICC cells incubated with culture supernatant from HCC cells (Fig. 6b). Hence, we have only focused on the effects of culture supernatant from ICC cells on HCC cells in our studies. Cancer cells with the mesenchymal phenotype show increased invasion and metastasis abilities and are associated with poor prognosis. The dismal prognosis of CHC is at least partially attributed to increased lymph node invasion 28. Our results from the wound healing and invasion assays demonstrate that the migration and invasion abilities of HCC cells are enhanced after incubated with culture supernatant from ICC cells. EMT of HCC cells induced by culture supernatant from ICC cells provides a possible mechanism for the aggressive behavior of CHC. Besides enhanced metastasis, EMT is also regarded as an important contributing factor to chemoresistance 29, 30. Our results show that culture supernatant from ICC cells could enhance the chemoresistance of HCC cells. Together these findings provide a new rationale for the poor prognosis of CHC.

*LAMC2* encodes the γ2 chain of laminin-332 and it is an important EMT-associated gene in various human cancers. For example, LAMC2 enhances the metastatic potential of lung adenocarcinoma through inducing EMT 15. Upregulated LAMC2 expression in prostate cancer cells induces EMT with enhanced migration 16. In cholangiocarcinoma, aberrant expression of LAMC2 correlates with invasion, metastasis, angiogenesis, and poor prognosis, which are attributed to EMT 31-33. A recent study has reported that laminin-332 sustains chemoresistance and supports cell 'stemness' in hepatocellular carcinoma 34. Studies from another report have demonstrated that laminin-332 induces EMT in hepatocellular carcinoma *in vitro* 18. Our results showed that the expression level of LAMC2 is higher in ICC cells but undetectable in HCC cells. After examining the level of LAMC2 in different culture supernatants, our studies have shown that LAMC2 is present in the culture supernatants of ICC cells and accumulates over a longer period (Fig. 5B, 24 h vs. 48 h). Furthermore, using a LAMC2 KO cell model, our studies have demonstrated that enhanced EMT of HCC cells induced by culture supernatants from ICC cells is attributed to LAMC2 secreted by those ICC cells. Both CHC and ICC show worse prognoses compared to HCC 1, 35, 36, and the prognosis of CHC is poorer than ICC 1. A previous study has pointed out that HCC will show aggressive phenotype with shorter recurrence-free and overall survival, once cholangiocarcinoma-like genes are expressed in these HCC cells 36. Consistent with that study 34, our results suggest that LAMC2 might be a cholangiocarcinoma-like gene causing a poor prognosis in HCC. Furthermore, our results have demonstrated that highly expressed LAMC2 is associated with increased mesenchymal phenotype in clinical CHC samples. These results suggest that promoting EMT of HCC cells by nearby ICC cells may contribute to the worse prognosis of CHC cases.

Collectively, our studies have demonstrated that cholangiocarcinoma cells, via the secretion of LAMC2, promote EMT of hepatocellular carcinoma cells, which may contribute to the poor prognosis of CHC. These findings provide new insight into the interaction of different types of liver cancer cells, which may help us find a new and effective therapy for CHC.

## Abbreviations

CHC: Combined hepatocellular cholangiocarcinoma
FBS: fetal bovine serum
LAMC2: Laminin subunit gamma 2
EMT: Epithelial-mesenchymal transition
ICC: Intrahepatic cholangiocarcinoma
HCC: Hepatocellular carcinoma
